# Receptor component protein, an endogenous allosteric modulator of family B G protein coupled receptors

**DOI:** 10.1016/j.bbamem.2019.183174

**Published:** 2020-03-01

**Authors:** Sarah J. Routledge, John Simms, Ashley Clark, Ho Yan Yeung, Mark J. Wigglesworth, Ian M. Dickerson, Philip Kitchen, Graham Ladds, David R. Poyner

**Affiliations:** a School of Life and Health Sciences, Aston University, Aston Triangle, Birmingham B4 7ET, UK; b Department of Pharmacology, University of Cambridge, Cambridge, CB2 1PD, UK; c Hit Discovery, Discovery Sciences, R&D, BioPharmaceuticals, AstraZeneca, Macclesfield, UK; d Del Monte Institute for Neuroscience, Department of Neuroscience, University of Rochester Medical Center, Rochester, NY 14642, USA

**Keywords:** AM, Adrenomedullin, CGRP, calcitonin gene-related peptide, CRF, corticotrophin releasing factor, CT, calcitonin, GIP, gastric inhibitory polypeptide, GLP, glucagon-like polypeptide, GPCR, G protein coupled receptor, HUVECs, Human umbilical vein endothelial cells, ICL, intracellular loop, RCP, receptor component protein, TM, transmembrane helix, Adrenomedullin, Accessory protein, GPCR signalling, CGRP, G protein coupling, Allosteric modulator

## Abstract

Receptor component protein (RCP) is a 148 amino acid intracellular peripheral membrane protein, previously identified as promoting the coupling of CGRP to cAMP production at the CGRP receptor, a heterodimer of calcitonin receptor like-receptor (CLR), a family B G protein-coupled receptor (GPCR) and receptor activity modifying protein 1 (RAMP1). We extend these observations to show that it selectively enhances CGRP receptor coupling to Gs but not Gq or pERK activation. At other family B GPCRs, it enhances cAMP production at the calcitonin, corticotrophin releasing factor type 1a and glucagon-like peptide type 2 receptors with their cognate ligands but not at the adrenomedullin type 1 (AM_1_), gastric inhibitory peptide and glucagon-like peptide type 1 receptors, all expressed in transfected HEK293S cells. However, there is also cell-line variability as RCP did not enhance cAMP production at the endogenous calcitonin receptor in HEK293T cells and it has previously been reported that it is active on the AM_1_ receptor expressed on NIH3T3 cells. RCP appears to behave as a positive allosteric modulator at coupling a number of family B GPCRs to Gs, albeit in a manner that is regulated by cell-specific factors. It may exert its effects at the interface between the 2nd intracellular loop of the GPCR and Gs, although there is likely to be some overlap between this location and that occupied by the C-terminus of RAMPs if they bind to the GPCRs.

## Highlights

• RCP promotes coupling of the CGRP receptor to Gs but not Gi or ERK activation.
• RCP enhances Gs coupling for the calcitonin, CRF 1a and GLP-2 receptors.
• RCP does not act on adrenomedullin 1, GIP or GLP-1 receptors in HEK293S cells.
• The actions of RCP depend on the cell line background.

## Graphical abstract

**Figure:**
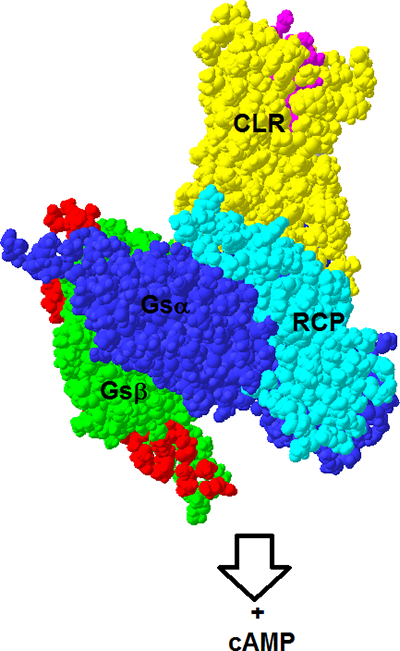

## 1 Introduction

RCP is a 148 amino acid, 17 kDa peripheral membrane protein and its expression has been demonstrated in numerous cell lines [1]. It is found in vivo in the brain, spinal cord, the uterus as well as in vasculature. It is a component of human RNA polymerase III where it is known as rpc9; homologues of this protein are found in yeast [2]. In addition to its role in RNA synthesis, RCP is also important in G protein-coupled receptor (GPCR) signalling. It is required for efficient coupling of the CGRP receptor to production of cAMP via Gs, the stimulatory G protein, as shown by knockdown of RCP expression [1,[3], [4], [5]]. CGRP receptors consist of a family B GPCR, the calcitonin receptor-like receptor (CLR). This requires an accessory protein, receptor activity modifying protein 1 (RAMP1) for ligand binding and receptor expression [6,7]. RCP is a third component of the receptor. It appears to physically associate with the receptor, interacting with its second intracellular loop (ICL2) [8]. In NIH3T3 cells, following challenge with CGRP, RCP translocates from the cell surface to the nucleus, perhaps suggesting a role in nuclear signalling in addition to facilitating Gs coupling [9]. Loss of RCP does not affect the affinity of CGRP for its receptor, or significantly alter trafficking to the cell surface, and so is not required in order for CLR and RAMP1 to interact [3]. Decreases in RCP expression have been correlated with reduced sensitivity to CGRP under a number of physiological and pathological conditions [1].

No effect is seen on the β_2_ adrenoreceptor or the adenosine 2A receptor following RCP knockdown, although the response of a peptide closely related to CGRP, adrenomedullin (AM), is also impaired by RCP knockdown in cells expressing its receptor (CLR in complex with RAMP2, a homologue of RAMP1) [3]. It remains an open question as to how far it can interact with other family B GPCRs.

There is growing awareness that receptors can be influenced allosterically by compounds or proteins that bind to locations distinct from the normal agonist binding site [10]. By enhancing or reducing the interaction of an intracellular allosteric modulator of a GPCR there is the potential to ‘tune’ the physiological response. Moreover, many GPCRs activate a wide array of downstream signalling pathways, depending on the activating agonist; this is biased agonism. Thus, allosteric modulators have the potential to influence GPCR signalling bias. This is seen with RAMPs, as they modulate ligand binding and signalling to a range of family B GPCRs [11]. It is possible that RCP is also an allosteric modulator at some family B GPCRs.

We are still far from understanding the pharmacological importance of RCP. Furthermore, no work has been done to systematically explore whether RCP interacts with other family B GPCRs. In this study, we investigate whether RCP is an allosteric modulator of CGRP receptor signalling, and determine whether its actions apply to other family B receptors; adrenomedullin 1, calcitonin (CT), glucagon-like polypeptide (GLP)-1, GLP2, gastric inhibitory polypeptide (GIP)-1 and corticotrophin releasing factor (CRF) type 1.

## 2 Materials and methods

### 2.1 Constructs

HA-tagged CLR, CTR, CRF1a, GLP-1R and FLAG-tagged RAMPs were as described previously [12,13]. The GIPR and GLP2R constructs were gifts from Dr. Simon Dowell (GSK, Stevenage, UK). All constructs were of human receptors.

### 2.2 Peptides

Human GLP-1 (7-36)NH_2_, GIP 1-42, and GLP2 (1-33)NH_2_ were synthesised by Alta Biosciences (University of Birmingham, Birmingham, UK). Human αCGRP, AM, CRF and CT were purchased from Bachem (Bubendorf, Switzerland). All peptides were made as 1 mM stocks in water containing 0.1% BSA.

### 2.3 Cell culture and transfections

HEK293S, HEK293T and SK-N-MC cells were cultured in Dulbecco's modified Eagles's medium supplemented with 10% fetal calf serum and 5% (v/v) penicillin/streptomycin (Invitrogen) in a humidified 95% air/5% CO2 atmosphere. Human umbilical vein endothelial cells (HUVECs) – pooled donor (PromoCell) were cultured in Endothelial Cell Growth Media (PromoCell) medium supplemented with 5% streptomycin (Invitrogen) in a humidified 95% air/5% CO2 atmosphere. Cells were plated onto 6 well, 24 well or 96 well plates for transfection. 30 nM siRNA (Invitrogen) was transfected per well with Lipofectamine 3000 reagent (ThermoFisher) according to the manufacturer's instructions to knock down endogenous RCP expression. 21-mer siRNA sequences to RCP were synthesised by Invitrogen: sense 5′-UCUGAAAGAGCAGCGUAAATT-3′ antisense 5′-UUUACGCUGCUCUUUCAG-3′. Control primers were scrambled sequences: UCUAGAAGACGUGCAGAAATT sense and UUU CUGCACGUCUUCUAGATT antisense.

DNA transfections were performed 48 h later, with 2 μg per well in a 6 well plate, 0.5 μg per well in a 24 well plate and 100 μg per well in a 96 well plate using Lipofectamine 3000 with P3000 reagent.

### 2.4 Cell surface expression determined by ELISA

ELISAs were performed in 24 well plates as previously [14] to determine cell surface expression of CLR and RAMPs in cells pretreated for 48 h with either scrambled siRNA or RCP siRNA before transfection with CLR and RAMP1.

### 2.5 Cell signalling assays

The cell signalling assays were performed as previously described [15]. Briefly, transiently transfected cells were harvested and washed with PBS. They were then resuspended in stimulation buffer (PBS containing 0.1% BSA and 0.5 mM isobutyl methylxanthine) and seeded at 3000 cells per well in 96-well white Optiplates. Ligands were added in the range of 1 pM to 1 μM and cAMP accumulation was measured after 8 min stimulation using LANCE® cAMP Detection Kit (PerkinElmer). Concentrations of cAMP were calculated using a cAMP standard curve performed in parallel. pERK was determined using 30,000 cells per well according to the manufacturer's instructions (CISBIO Phospho-ERK (Thr202/Tyr204) kits) and normalised to phorbol 12-myristate 13-acetate. Intracellular calcium experiments were performed by seeding cells into 96 well plates and growing in Dulbecco's modified Eagles's medium supplemented with 10% fetal calf serum and 5% (v/v) penicillin/streptomycin. 48 h post transfection, media was replaced with Hanks buffered-saline without calcium. Intracellular calcium was then measured using a Fluo-4 Direct Calcium Assay kit (Thermo Fisher Scientific) upon stimulation with ligand, and values were normalised to ionomycin. All assays were read using a Mithras LB 940 plate reader (Berthold Technologies).

### 2.6 RT-PCR details

Media was removed from cells in a 6 well plate and cells were washed twice (PBS). Approximately 2 × 10^5^ cells were used per extraction. RNA extraction was performed using RNAqueous-4PCR kit (Ambion). Complementary DNA was synthesised using a QuantiTect reverse transcription kit (Qiagen) and used in real-time PCR with primers (Table 1) synthesised and supplied by Sigma Aldrich. Electrophoresis was performed on a 2% agarose gel. The image was captured using G Box iChemi gel documentation system and densitometry analysis performed using GeneTools (Syngene). mRNA levels of the genes of interest were normalised to GAPDH expression.

**Table 1:** Primers used for PCR.

Oligonucleotides	Sequence	Amplicon size (bp)
Human		
GAPDH	5′ - TGCACCACCAACTGCTTAGC- 3′	87
	5′ - GGCATGGACTGTGGTCATGAG- 3′	
CLR	5′ - ACCAGGCCTTAGTAGCCACA - 3′	298
	5′ - ACAAATTGGGCCATGGATAA - 3′	
RAMP1	5′ - CTGCCAGGAGGCTAACTACG - 3′	298
	5′ - GACCACGATGAAGGGGTAGA - 3′	
RAMP2	5′ - GGGGGACGGTGAAGAACTAT - 3′	227
	5′ - GTTGGCAAAGTGGATCTGGT - 3′	
RAMP3	5′ - AACTTCTCCCGTTGCTGCT - 3′	353
	5′ - GACGGGTATAACGATCAGCG - 3′	
RCP	5′ - AGAGCAGCGTAAAGAAAGTGG - 3′ 5′ - CTGACAATTTCAGGACTCTGGTG - 3′	129

### 2.7 Data analysis

Concentration-response curves were analysed via PRISM 7.0e (San Diego, CA). Data were fitted to obtain concentration-response curves using the three-parameter logistic equation for pEC_50_ and Emax values. Data was normalised according to the fitted basal and maximal responses for the RCP scrambled antisense control curves. Statistical analysis was performed using a Student's *t*-test or one-way ANOVA with Bonferroni's or Dunnett's multiple comparisons as appropriate.

### 2.8 Molecular modelling

A molecular model of the RCP/CLR/RAMP1/Gαsβγ complex was built using the cryo-EM structure of CLR/RAMP1/Gαsβγ (PDB ID: 6E3Y), and a homology model of RCP built in Modeller using the yeast homologue C17 as template (PDB ID: 3AYH). ICL2 was previously identified as the RCP contact site [8]; we therefore placed the RCP model at a distance of 5 Å from the centre of ICL2 “outwards” from the receptor core, and docked RCP locally using the ROSIE webserver, followed by sidechain refinements using Rosetta. In situ, the CLR/RAMP1 complex would be embedded in a lipid bilayer, therefore binding poses which were not compatible with this (i.e. with significant hydrophilic/charged portions of RCP extending into the membrane core) were discarded as unphysical.

## 3 Results

### 3.1 Expression of RCP and other receptor components

**Fig. 1:**
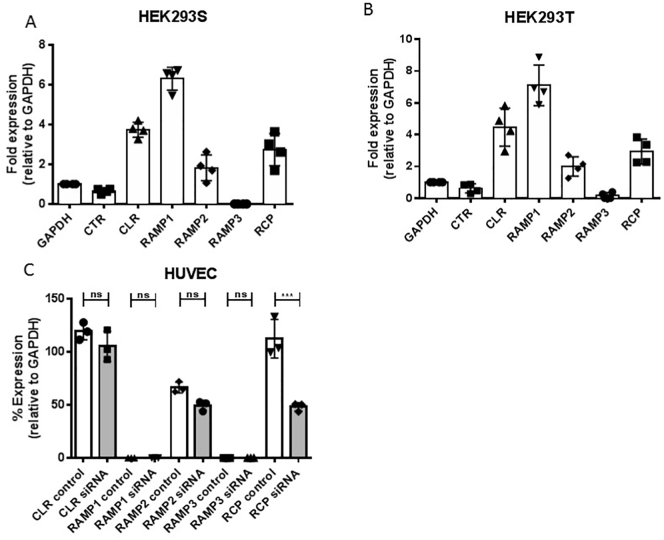
Expression of receptor components and their selectively knocked down by siRNA. A) Expression of receptor components by RT-PCR in HEK293S cells; B) Expression of receptor components by RT-PCR in HEK293T cells; C) Expression of receptor components by RT-PCR in HUVEC cells with the effect of pretreatment with 100 nM RCP siRNA to 100 nM scrambled siRNA for 48 h. Statistical testing was by Student's t. Means, s.e.ms and individual data points are illustrated.

We initially investigated the distribution of RCP and potential partners in HEK293S, HEK293T and HUVEC cells (Fig. 1a, b and c). All three cells expressed RCP and CLR; the HEK293S and T cells had additionally RAMPs 1 and 2 whereas HUVECs expressed only RAMP2. The specificity of the siRNA construct against RCP was shown in HUVECs, where it only reduced RCP expression (Fig. 1c). Furthermore, in HEK293S cells, cell surface ELISA confirmed that expression of HA-CLR in the presence of RAMP1 was unaffected by siRNA to RCP (135 ± 35% of control treated with scrambled siRNA, *n* = 3).

### 3.2 RCP is an allosteric modulator of CLR that influences agonist bias

**Fig. 2:**
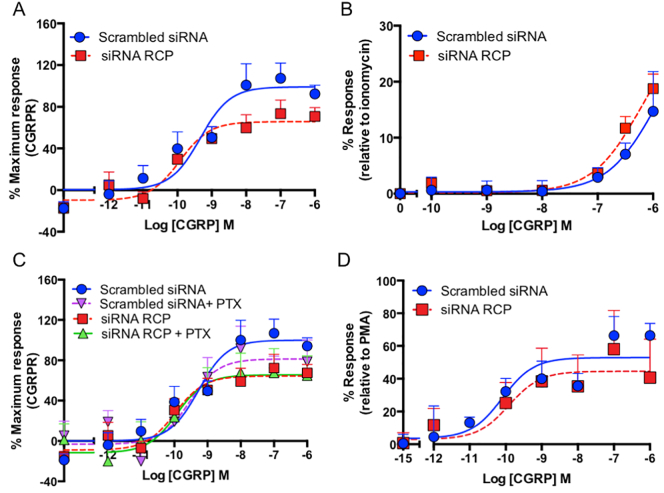
RCP specifically influences agonist bias at the CGRPR by selectively enhancing coupling to Gs. In transfected HEK293S cells CGRP-mediated cAMP production is impaired by RCP knockdown (A) but not Ca^2+^ mobilisation (B), pertussis toxin sensitivity (C) or activation of pERK (D). In all experiments *n* = 3 ± S.E.M.

Further investigations showed that CGRP stimulated cAMP production (Gs) (pEC_50_ 9.34 ± 0.27), pERK phosphorylation (pEC_50_ 10.09 ± 0.40) and increased intracellular calcium mobilisation (Gαq) (pEC_50_ 5.96 ± 0.30); in the presence of pertussis toxin there was no significant increase in CGRP potency on cAMP production (Fig. 2), suggesting that in these cells, there was little or no coupling to Gi. Whilst there were no differences in the responses in the presence of siRNA to knock down RCP for pERK, calcium, or pertussis toxin, for cAMP the E_max_ was reduced by 33.5 ± 2.9% (*P* < .001, Student's t) with no change in the pEC_50_ (Fig. 2).

### 3.3 RCP interacts with a subset of family B GPCRs

**Fig. 3:**
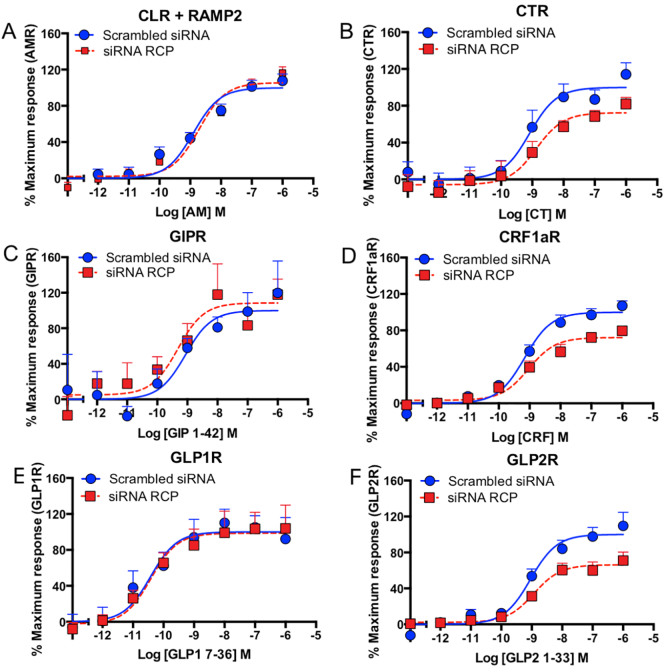
RCP knockdown impairs cAMP production at other family B receptors. In transfected HEK293S cells, cAMP production was attenuated at the CTR (B), CRF1aR (D) and GLP2R (F) with their cognate ligands but not at the AM receptor (A), GIPR (C) and GLP1R (E). In all experiments, *n* = 3 ± S.E.M.

Current pharmacological studies point to a direct interaction with intracellular loops 2 (ICL2) of CLR [8]. For Family B GPCRs, there is a high degree of sequence conservation within this region, and could therefore indicate possible interactions of RCP with other family B receptors. We therefore investigated whether knock down of RCP affects signalling of other family B receptors. We observed impairment of the maximum cAMP production (but not pEC_50_) in transfected HEK293S cells by CT at the CTR (100.00 ± 4.95 to 72.47 ± 7.09, *P* = .0319), CRF at the CRF1a receptor (100.00 ± 3.87 to 72.23 ± 3.39, P = .0319) and GLP-2 at the GLP-2R (100.00 ± 6.04 to 66.16 ± 4.13, *P* = .029) (Fig. 3, Table 2). For the AM_1_ receptor, GIPR and GLP1R, there were no significant changes to E_max_ or pEC_50_.

**Table 2:** E_max_ and pEC_50_ values for GPCR cAMP RCP knockdown assays.

GPCR	Peptide	Scrambled siRNA E_max_	siRNA RCP E_max_	Scrambled siRNA pEC_50_	siRNA RCP pEC_50_
CLR + RAMP1	CGRP	100.00 (±4.33)	73.33 (±2.88)^⁎⁎⁎^	8.97 (±0.13)	8.76 (±0.13)
CLR + RAMP2	AM	100.00 (±4.95)	105.70 (±4.69)	8.90 (±0.15)	8.72 (±0.14)
CRF1aR	CRF	100.00 (±3.89)	72.47 (±7.09)**	9.16 (±0.12)	9.03 (±0.14)
CTR	CT	100.00 (±6.04)	72.47 (±7.09)*	9.09 (±0.25)	8.87 (±0.26)
GIPR	GIP 1–42	99.99 (±17.43)	108.60 (±13.53)	9.07 (±0.55)	9.32 (±0.42)
GLP1R	GLP1 7–36	100.00 (±8.66)	98.53 (±8.32)	10.43 (±0.33)	10.41 (±0.33)
GLP2R	GLP2 1–33	100.00 (±6.04)	66.16 (±4.13)*	9.043 (±0.17)	8.93 (±0.19)

All values are *n* > 3, ±S.E.M. *, **, ****, *P* < .05, 0.01, 0.001, Student's t. Values are normalised to cAMP seen with 10 μM forskolin.

### 3.4 RCP action on endogenously expressed CGRP and AM_1_ receptors

**Fig. 4:**
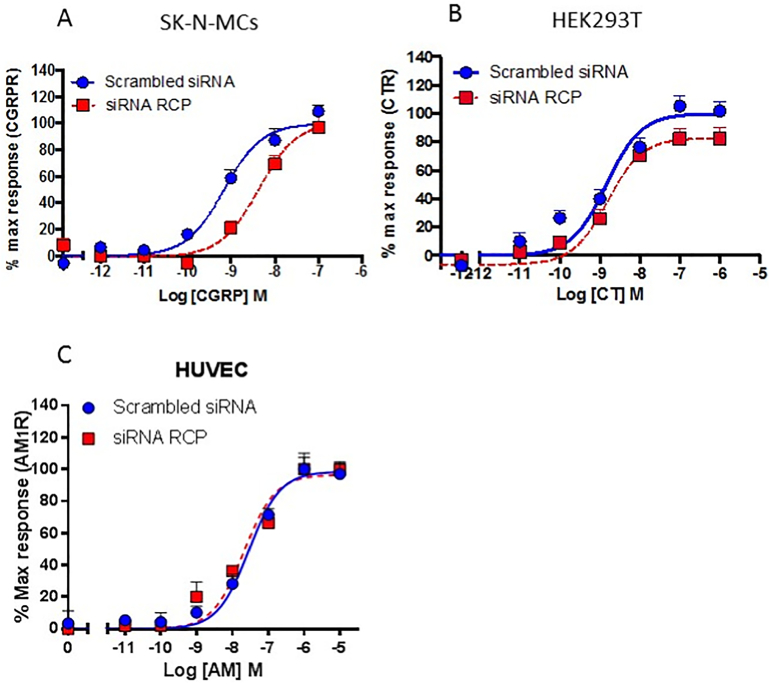
Effects of RCP knockdown on Gs coupling of endogenous receptors. cAMP levels were determined from A; SK-N-MC B; HEK293T and C; HUVECS when stimulated with agonists (CGRP in A), CT in B) and AM in C) in the presence or absence of siRNA to RCP. For each data set, n = at least 3 ± S.E.M.

We next investigated whether RCP knock down affected the signalling of endogenously expressed family B receptors in SK-N-MC [16], HEK293T [12] and HUVEC [17] cells. RCP knockdown by siRNA reduces agonist stimulated cAMP production by endogenously expressed CGRPR in SK-N-MC cells (Fig. 4a). There was no significant change to E_max_ (albeit this may not be robustly defined as it is relies only on data from one point) but there was a significant change to pEC_50_ upon RCP knockdown (pEC_50_ 9.16 ± 0.12 to 8.37 ± 0.13, *P* < .0001). We observed marginal effects upon the CTR expressed by HEK293T cells (Fig. 4b), and no effects upon the AM_1_ receptor in HUVECs (Fig. 4c), although both these cell lines express RCP (Figs. 1b, c).

### 3.5 Molecular modelling of RCP and its interactions with CLR/RAMP1

**Fig. 5:**
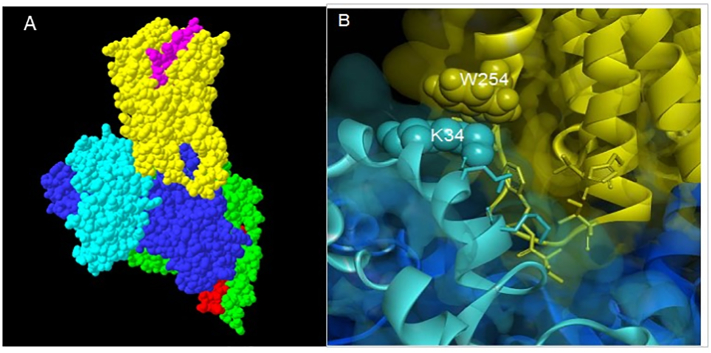
Molecular model of the RCP in complex with RAMP1-CLR. A; Model of RCP (light blue), CLR (yellow), CGRP (purple), Gαs (dark blue) Gβ (green), Gγ (red). B; Interface between ICL2 of CLR (yellow), RCP (light blue) and Gαs (dark blue), marking Lys34 and Trp254.

As there is evidence that RCP interacts with TM3/ICL2 of CLR [8], we carried out docking simulations of RCP (modelled using the yeast homologue C17, PDB ID: 3AYH) to the transmembrane domain of CLR, with both the N-terminus of CGRP and the trimeric Gαsβγ bound (from the cryo-electron microscope structure of this complex, PDB ID: 6E3Y). The best fit of RCP was to a surface formed by ICL2 of CLR and Gαs (Fig. 5). This included the TM3/ICL2 peptide of CLR previously identified as the RCP contact site [8]. The Gαs interface with RCP involves the αB-αC loop that shows little homology to other G proteins. Within RCP, Lys24, Lys28 and Lys32 formed a strongly basic patch making electrostatic interactions with CLR and Gs and Lys32 would be able to interact with Trp254 of CLR. This Trp is also found in the calcitonin, CRF and the Drosophila CG174154 receptors, although in the GLP-2R it is an arginine. The CLR interface also overlaps with the interaction site between the C-terminus of RAMP1 and CLR [18]. In order to accommodate RCP, it is likely that there would have to be significant distortion of the C-terminus of the RAMP. Unfortunately, the residues at the extreme C-terminus of the RAMP are not resolved in the available structure (ie PDB ID: 6E3Y, making it hard to plausibly model. However, a role for the C-terminus of the RAMP would provide an explanation for why, in our hands, the CGRP (CLR/RAMP1) but not AM (CLR/RAMP2) receptors show a robust interaction with RCP.

## 4 Discussion

In this paper we have demonstrated that RCP acts on the CGRP receptor to selectively augment coupling to Gs/cAMP but not Gq/Ca^2+^ or pERK activation. The lack of significant Gi coupling observed in control cells makes it hard to say whether this is influenced by RCP but we saw no signs of any augmentation after RCP knock-down. We have previously noted a lack of Gi coupling at the CGRP receptor in HEK293 cells [15]. RCP can also promote cAMP accumulation in transfected HEK293S cells at the CT, CRF and GLP2 receptors but not for the AM1, GIPR and GLP1 receptors. It is clear the RCP is of more general significance for family B GPCRs than previously thought, potentially acting as an allosteric modulator to promote Gs coupling. Interestingly, a Drosophila family B GPCR, CG17415, also appears to interact with a homologue of RCP and human RCP can enhance coupling of this receptor to Gs. Thus RCP-receptor interactions may have co-evolved with the emergence of family B GPCRs [19]. The physiological implications of an increased role for RCP remain to be documented.

We found variations in the response to RCP as its knock-down in HEK293T cells expressing an endogenous calcitonin receptor (as seen in Fig. 4b) had little effect on cAMP, unlike transfected cells. Further, whilst we observed no effect of RCP knockdown on the responsiveness of the AM_1_ receptor in transfected cells or HUVECs endogenously expressing the receptor, in NIH3T3 cells, the activation of an endogenous AM_1_ receptor was impaired when these were modified to express an antisense RNA directed against RCP [5]. The dependence of RCP on cell line recalls similar variability seen with RAMPs [12,20]. In both cases, this is likely to be due to differences in signalling components between cell lines. Differences in receptor expression (by changing receptor reserve) will particularly influence the sensitivity of the maximum response to modulators. With a large receptor reserve, a decrease in receptor function will appear as a change in EC_50_ whereas if there is no reserve, then an effect on maximum response would be apparent. However, in the current study, we see a decrease in maximum response in transfected cells where receptor it might be expected that there is a large receptor reserve assuming efficient transfection. Other factors such as scaffolding proteins and access to G proteins might be important, as well as translational regulation of receptor components, only functional in cells endogenously expressing the relevant receptor. Furthermore, reversible covalent modifications such as phosphorylation could potentially play a role in governing RCP-receptor interactions. Thus these may be subject to dynamic control, an interesting concept given evidence that RCP is also translocated to the nucleus after CLR activation [9].

To fully appreciate the physiological significance of these results, it will first be important to examine the co-distribution of receptors and RCP at a cellular level in tissues, or at least in primary cells derived from tissues. Lack of this information has greatly hindered the physiological understanding of RAMPs [21]. However, RCP has the potential to accentuate cAMP signalling over other pathways and this will be particularly significant where the latter are associated with deleterious effects [22].

In conclusion, we have demonstrated that RCP can act as a positive allosteric modulator, promoting the coupling of specific family B receptors to Gs. Future work could usefully be directed at understanding the significance of this.

## Funding

This work was supported by the Biotechnology and Biological Sciences Research Council [grant number BB/M000176/2] awarded to GL and DRP. Data supporting this paper is deposited in the Aston University research repository at 10.17036/researchdata.aston.ac.uk.00000451. We also acknowledge the support of the Rosetrees foundation (to HYY and GL). HYY is also supported by an international scholarship from the Cambridge Trust. PK is supported by an Aston University 50th Anniversary Prize Fellowship. We acknowledge the use of Athena at HPC Midlands+, which was funded by the EPSRC on grant EP/P020232/1, in this research, as part of the HPC Midlands+ consortium.

## Transparency document

Transparency document. Image 1

## Declaration of competing interest

M.W. is an employee of and shareholder in AstraZeneca. The remaining authors have no competing interests.
